# Evaluating the Empowerment Potential of an International Sexual Support Website for Patients with Anorectal Malformations and Hirschsprung Disease, their Parents and Healthcare Providers

**DOI:** 10.1055/a-2635-7802

**Published:** 2025-07-09

**Authors:** Olivia K.C. Spivack, Irene K. Schokker-van Linschoten, Marjolein Spoel, Annette Lemli, Dalia Aminoff, Mikko Pakarinen, Ivo de Blaauw, Hanneke Ijsselstijn, Violet Petit-Steeghs

**Affiliations:** 1 Department of Pediatric Surgery, Erasmus MC Sophia Children's Hospital, Rotterdam, the Netherlands; 2 SoMA e.V. - Selbsthilfeorganisation für Menschen mit Anorektalfehlbildungen und Morbus Hirschsprung - German Patient Organization for Anorectal Malformations and Hirschsprung disease, Munich, Germany; 3 AIMAR - Associazione Italiana Malformazioni Anorettali – OdV - Italian Patient Organization for Anorectal Malformations, Rome, Italy; 4 Department of Pediatric Surgery, New Children's Hospital, University of Helsinki and Helsinki University Hospital, Pediatric Liver and Gut Research Group, Helsinki, Finland; 5 Division of Pediatric Surgery, Department of Surgery, Radboudumc-Amalia Children's Hospital, Nijmegen, the Netherlands; 6 Erasmus School of Health Policy and Management, Erasmus University Rotterdam, Rotterdam, the Netherlands

**Keywords:** ERNICA, ERN eUROGEN, European Reference Network, rare disease, digital health

## Abstract

**Introduction:**

Research indicates that the sexual support needs of patients with anorectal malformations (ARM) and Hirschsprung disease (HD) are often not addressed by patients, parents, and healthcare professionals (HPs) in their interactions. An international support website was developed to empower stakeholders, by addressing identified barriers. This study aimed to explore the empowerment potential of this disease-specific tool.

**Materials and Methods:**

Two online surveys were disseminated between May 1 and October 1, 2023; one for HPs and another for patients/parents. The surveys sought to assess and understand the website's expected empowerment effect. Empowerment was conceptualized using patient/professional empowerment models. Data were descriptively analyzed.

**Results:**

A total of 12 patients (ARM, *n* = 11; HD, *n* = 1), 17 parents (ARM, *n* = 9; HD, *n* = 8), and 20 HPs responded to the survey. HPs largely expected the website to have a positive empowerment effect, by providing a sense of meaning, information, support, and opportunities to learn and grow. Less of an effect was expected for “freeing up resources.” For patients and parents, an empowerment effect was also expected, by generating the knowledge, skills, attitudes, and self-awareness necessary to influence their own behavior and by providing a sense of meaning and coherence. Respondents experienced the website positively, yet one patient and one parent considered the website “fully complete.” Inclusivity, cultural sensitivity, and accessibility were highlighted as focus points.

**Conclusion:**

To increase the website's empowerment potential, attention should be paid to inclusivity, cultural sensitivity, and accessibility, as well as its implementation within the (institutional) contexts where patients, parents, and HPs interact.

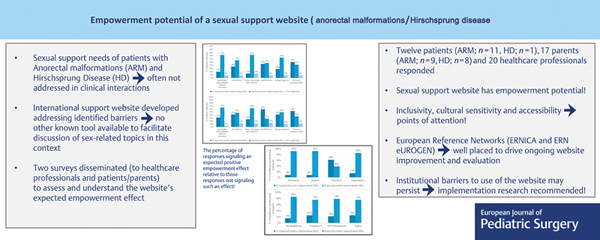

## Introduction

Anorectal malformations (ARM) and Hirschsprung disease (HD) are rare congenital colorectal anomalies. Most children born with ARM or HD are surgically treated in early life. However, throughout the life course, they may encounter difficulties such as fecal 1 2 3 4 5 6 and urinary 2 7 8 incontinence, constipation, 2 3 5 6 9 sexual dysfunction, 1 2 7 10 11 12 and dyspareunia. 13 14 Appearance of the external genitalia may be affected and individuals may have a stoma and/or postoperative scarring. Sexual well-being 15 and relationships are often compromised as a result. 4 9 11 14 16 17 18 19 20 For many patients, fertility, pregnancy, and childbirth are also topics of concern. 16 17 19 21

Research indicates that sexual support needs are often not addressed by patients with ARM/HD, 19 their parents, 21 and healthcare professionals 12 in their interactions. This may be the result of a range of factors, such as difficulties broaching the topic, and having a lack of time, knowledge, and awareness. 22 With sexual development beginning in childhood and continuing through adolescence and adulthood, providing sexual support is key to fostering a holistic transitional pathway from pediatric to adult care. 23 24 Patients and parents have voiced a desire for pediatric surgeons, among others, to address sex-related topics. 9 21 23 Although pediatric surgeons have recognized their responsibility to do so, they often do not feel equipped. 25

Various tools have been developed to facilitate communication with young people about sex-related topics. 26 27 28 Some have been developed specifically for those with chronic conditions. 27 28 However, members of the Dutch consortium “Support Psychosexual problems Congenital Colorectal malformations” considered their applicability to be limited in the context of ARM/HD, with healthcare professionals finding it difficult to integrate the tools in practice. 22 A sexual support website was therefore developed (in Dutch), aligned to the specific needs of patients with ARM/HD and their healthcare providers. 22 By addressing identified barriers, the website aimed to empower patients with ARM/HD, their parents, and healthcare providers in the Netherlands to address relevant sex-related topics in their interactions. 22 Information and resources specific to the Dutch healthcare context were included. Models informing the development of other existing sex-related communication tools 27 29 were also used to develop website content.

To increase visibility of the website, the European Reference Network (ERN) for Rare Inherited Congenital Anomalies (ERNICA) 30 and the European Reference Network for Rare Urogenital Diseases and Complex Conditions (ERN eUROGEN) 31 assumed responsibility for its management in 2023 ( https://www.seksualiteit-arm-zvh.nl/ ). To increase accessibility of the support tool, an English version tailored to an international audience was developed ( https://www.sexuality-arm-hd.com/ ). The Dutch version was also updated with relevant, internationally developed resources. An initial evaluation of the original Dutch website revealed that patients and healthcare professionals expected the tool to have a positive effect on their empowerment, fostered by promoting a collaborative dialogue, stimulating awareness, closing knowledge gaps, and showing possibilities for support. 22 Although we expect similar results for the Dutch–English ERNICA/eUROGEN sexual support website, the tool has not yet been evaluated.

The aim of this study was therefore to evaluate the extent to which the ERNICA/eUROGEN website is expected to have an empowerment effect, among patients with ARM/HD, parents, and healthcare professionals internationally. In doing so, this explorative study aimed to shed light on the empowerment potential of this digital tool in empowering stakeholders to address sex-related topics in their interactions.

## Methods

### Study Design

This study had an observational, cross-sectional design and was a collaboration between ERNICA and ERN eUROGEN. European Reference Networks (ERNs) are networks of specialized hospitals and patient organizations seeking to pool together disease-specific expertise from across Europe. 32 While HD falls within the clinical scope of ERNICA, ARM is covered by ERN eUROGEN. An ERNICA–eUROGEN working group exists to facilitate cross-ERN collaboration.

### Website Evaluation Surveys

Two anonymous surveys were developed using the online EUSurvey platform: (1) for healthcare professionals involved in the care of ARM/HD and (2) for patients with ARM/HD and their parents.

#### Survey for Healthcare Professionals Involved in the Care of ARM/HD

This survey was comprised of three parts. Part A was designed to explore the profiles of website users, collecting demographic data on participants' professional position, place of work, biological sex, and age category.

**Fig. 1:**
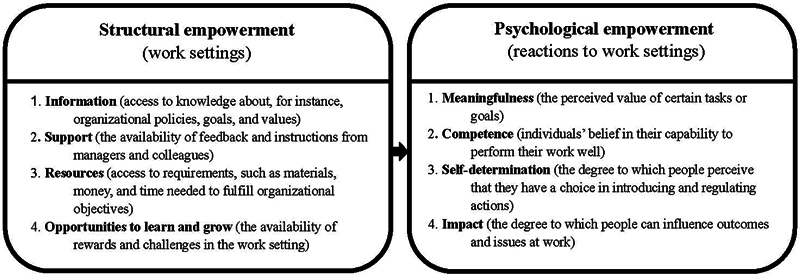
What professionals need to become empowered: Structural empowerment model of Kanter (1987) 33 and psychological empowerment model of Thomas and Velthouse (1990), 34 as summarized by Petit-Steeghs et al. (2020). 22

Part B was designed to assess the website's expected empowerment effect. Participants were asked to rate the extent to which they agreed or disagreed with 24 statements using a 5-point Likert scale (strongly disagree, disagree, neutral, agree, strongly agree, non-applicable). These statements, formulated on the basis of structural 33 and psychological 34 empowerment models, were drawn from those employed by Petit-Steeghs et al 22 in their evaluation of the original Dutch website in the Netherlands. The key components of these models are depicted in Fig. 1 . See Supplementary File 1 (available in the online version) for an overview of Part B statements aligned to their relevant empowerment component.

Part C, comprised of 20 questions, was designed to explore users' experience of the website. This information was collected to give context to the (expected) empowerment effect.

#### Survey for Patients with ARM/HD and Their Parents

Part A was designed to explore the profiles of website users. Participants were asked to indicate whether they were a patient born with ARM or HD or a parent of a child. Further information was collected (from all respondents) on the patients' condition, age, country of residence and birth, childhood environment (rural/urban), biological sex, receipt of medical care (and bowel irrigations specifically), presence of a stoma, sexual activity, and experience of sexual problems (past and present).

**Fig. 2:**
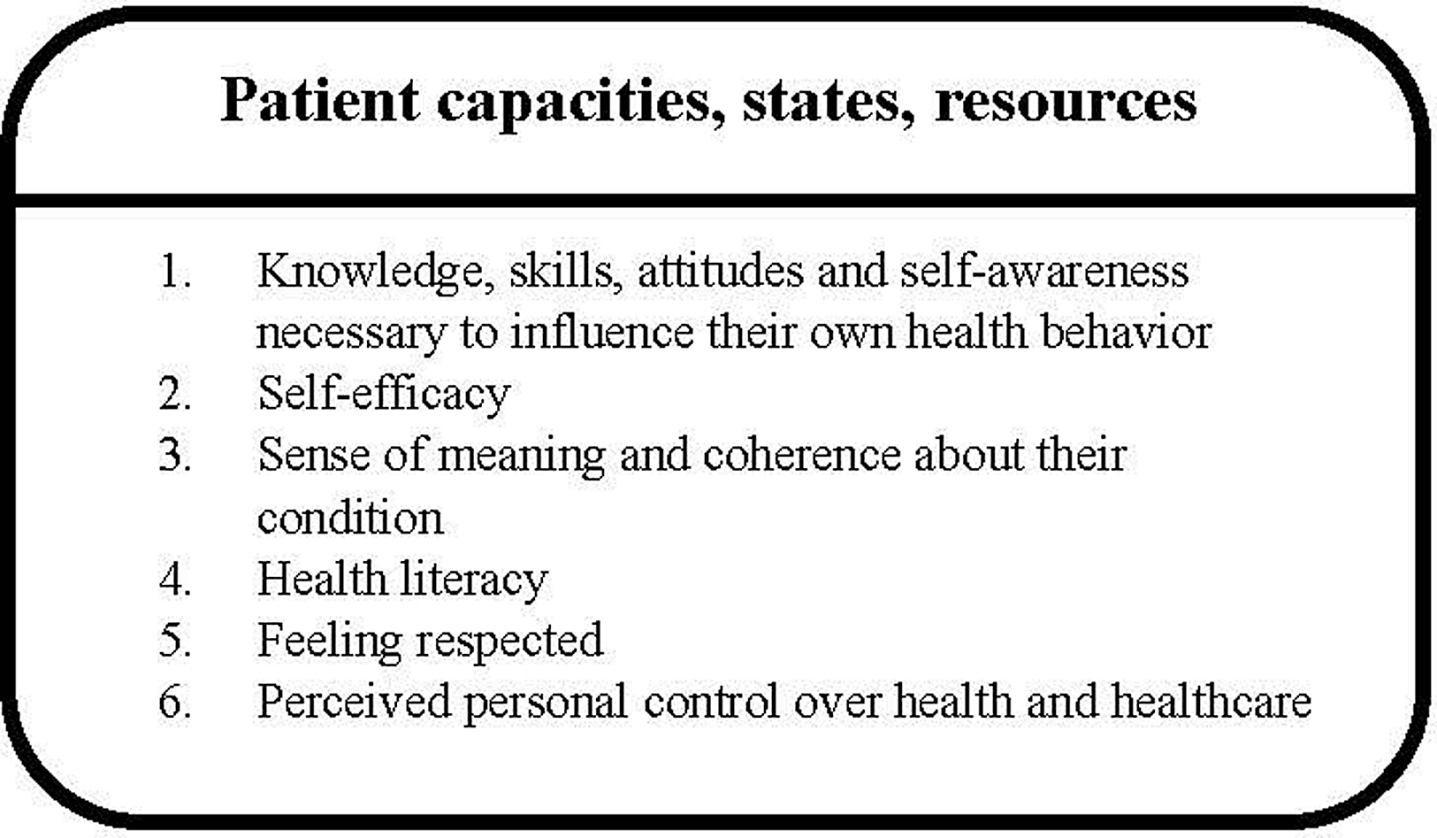
What patients need to become empowered: Patient empowerment model of Bravo (2015), 35 as summarized by Petit-Steeghs et al. (2020). 22

Part B was designed to assess the website's expected empowerment effect. Patients and parents were asked to rate the extent to which they agreed or disagreed with 18 statements using a 5-point Likert scale (strongly disagree, disagree, neutral, agree, strongly agree, non-applicable). These statements, formulated on the basis of a patient empowerment model, 35 were also drawn from those employed by Petit-Steeghs et al. 22 The key components of this model are depicted in Fig. 2 . See Supplementary File 1 (available in the online version) for an overview of Part B statements aligned to their relevant empowerment component.

Part C, comprised of 19 questions, was designed to explore users' experience of the website. This information was also collected to give context to the (expected) empowerment effect.

### Dissemination

To promote online survey participation, the ERNICA and ERN eUROGEN project management teams circulated information on the sexual support website and its evaluation within their respective ERNs via email. Healthcare professionals and representatives from relevant patient organizations were specifically requested to disseminate the website (and its evaluation) within their respective communities. The ERNICA/eUROGEN newsletters, websites, and social media channels were also used for dissemination purposes. Furthermore, individuals involved in the original development of the (Dutch) sexual support website were contacted and asked to promote participation.

Dissemination efforts took place within a 5-month data collection period, between May 1 and October 1, 2023. All patients with ARM or HD, their parents, and healthcare professionals were encouraged to participate. Participants could access their respective evaluation survey in 24 different European languages, facilitated through the EUSurvey's machine translation function (see Supplementary File 2 (available in the online version) for an overview of available languages).

### Ethical Approval

The Medical Ethical Committee at Erasmus University Medical Center reviewed the study protocol, subsequently confirming its approval (IRB approval MEC-2022-0638 and MEC-2022-0639). Data were collected and analyzed anonymously and stored securely on the EUSurvey platform and in a protected part of the Erasmus MC server. Informed consent was obtained from survey participants. Healthcare professionals' disclosure of potentially identifiable information (hospital name) was optional.

### Data Analysis

Survey data was descriptively analyzed using Microsoft Excel. Absolute and relative (%) values were calculated for each survey question. For participants' age, the median and range were calculated. To analyze the expected empowerment effect, the absolute and relative (%) number of responses signaling a positive effect were assessed for each empowerment component as displayed in Figs. 1 and 2 . “Agree” and “strongly agree” were considered responses signaling a (potential) positive empowerment effect. “Neutral,” “disagree,” or “strongly disagree” indicated no positive empowerment effect.

## Results

### User Profiles

Surveys were completed by 20 healthcare professionals, 12 patients, and 17 parents.

**Fig. 3:**
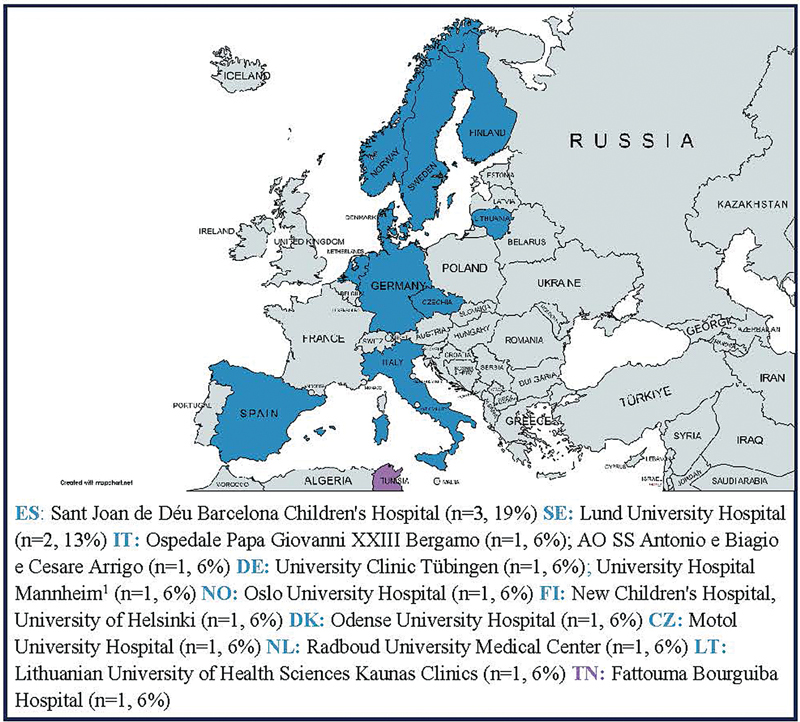
Overview of hospital locations. Abbreviations: ES, Spain; SE, Sweden; IT, Italy; DE, Germany; NO, Norway; FI, Finland; DK, Denmark; CZ, Czech Republic; NL, the Netherlands; LT, Lithuania; TN, Tunisia. Countries colored in blue are a part of the European Union/European Economic Area [EU/EEA], countries colored purple are not. Percentages are rounded to the nearest whole number. As a result, they may not add up to 100%. 1 This participant responded with ‘Mannheim’ only - the University Hospital Mannheim is assumed.

All healthcare professionals worked in hospital-based settings ( *n* = 20, 100%), with those disclosing their place of work ( *n* = 16, 80%) situated predominantly in countries part of the European Union/European Economic Area (EU/EEA) ( *n* = 15, 94%). See Fig. 3 for an overview of hospital locations. The majority of healthcare professional respondents were pediatric surgeons ( *n* = 15, 75%), with one identifying as a pediatric surgeon-urologist.

Most patients who completed the survey were born with ARM ( *n* = 11, 92%). The median age was 31 years (range 21–65) and most of them were biologically female ( *n* = 7, 58%). Patients resided in one of five countries, both within ( *n* = 6, 50%) and outside ( *n* = 6, 50%) the EU/EEA. Most patients reported being or having been sexually active ( *n* = 11, 92%), with 7 (64%) disclosing past sexual problems and the remaining 4 (36%) facing problems in both the past and present.

**Fig. 4:**
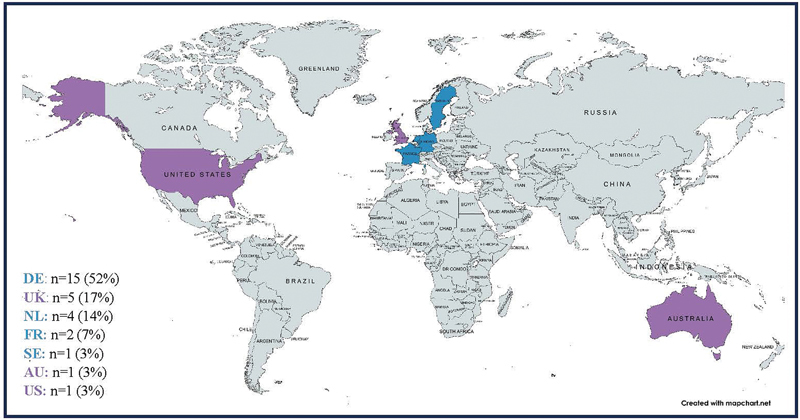
An overview of patients' residing countries (as reported by patients themselves and their parents). Abbreviations: DE, Germany; UK, United Kingdom; NL, the Netherlands; FR, France; SE, Sweden; AU, Australia; US, United States of America. Countries colored in blue are a part of the European Union/European Economic Area [EU/EEA], countries colored purple are not. Percentages are rounded to the nearest whole number. As a result, they may not add up to 100%.

Parents of biologically male ( *n* = 9, 53%) and female ( *n* = 8, 47%) patients born with ARM ( *n* = 9, 53%) or HD ( *n* = 8, 47%) completed the survey. Their children had a median age of 12 years (range 3–28). Patients were reported to reside in one of five countries, predominately situated in the EU/EEA ( *n* = 16, 94%). See Fig. 4 for an overview of patients' country of residence (as reported by patients themselves and their parents). Most parents reported that their child was not and had not been sexually active in the past ( *n* = 14, 82%). For those who considered their child to have sexual experience ( *n* = 2), one (with ARM) was reported to have past sexual problems and for the other, this was unknown.

See Supplementary File 3 (available in the online version) for additional user information.

### Expected Empowerment Effect

#### Healthcare Professionals Involved in the Care of ARM/HD

**Fig. 5:**
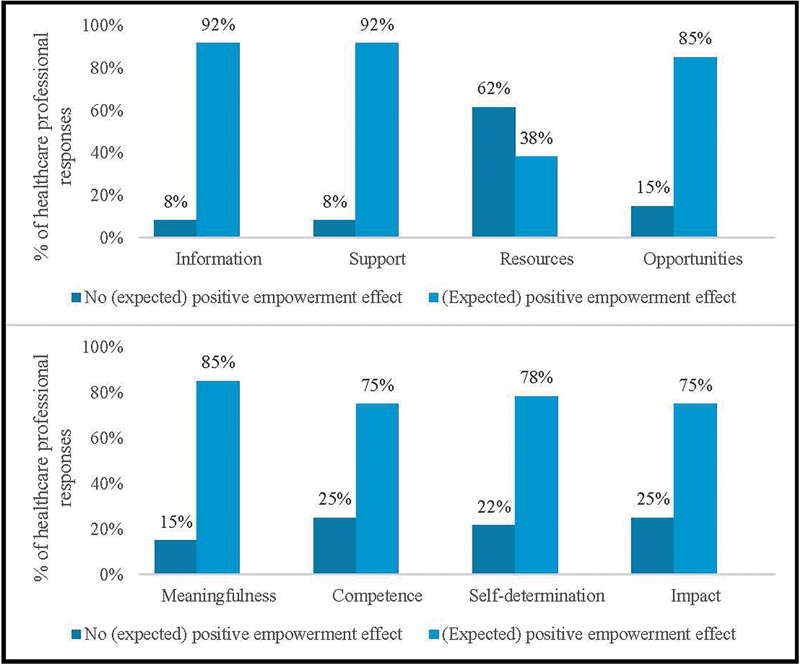
The percentage of (healthcare professional) responses signaling an expected positive empowerment effect relative to those responses not signaling such an effect – grouped into the components of professional empowerment models Kanter (1987) 33 and Thomas and Velthouse (1990). 34 No respondent answered ‘Non-applicable’ to any question.

Healthcare professionals largely expected the website to have a positive effect on their structural empowerment, by providing information (92%), support (92%), and opportunities to learn and grow (85%). However, their expectations concerning the website's potential in enhancing access to resources were somewhat lower, with most responses indicating no expected empowerment effect in this area (62%). Respondents also expected the website to have a positive effect on their psychological empowerment, fostered by generating an increased sense of meaning; “meaningfulness” (85%), competence (75%), self-determination (78%), and a perceived ability to provide impact (75%). Fig. 5 depicts the percentage of responses signaling an expected positive empowerment effect, relative to those responses not signaling such an effect.

#### Patients with ARM/HD

Patients largely expected the website to have a positive effect on their empowerment, by generating the knowledge, skills, attitudes, and self-awareness necessary to influence their own health behavior (72%), a sense of meaning and coherence about their condition (72%), and feelings of respect (64%). Less of a positive effect was expected for self-efficacy (56%), health literacy (47%), and perceived personal control (42%). Notably, for perceived personal control, responses signaling “no expected positive empowerment effect” (50%) outnumbered those signaling any “positive effect.”

#### Parents of Children with ARM/HD

**Fig. 6:**
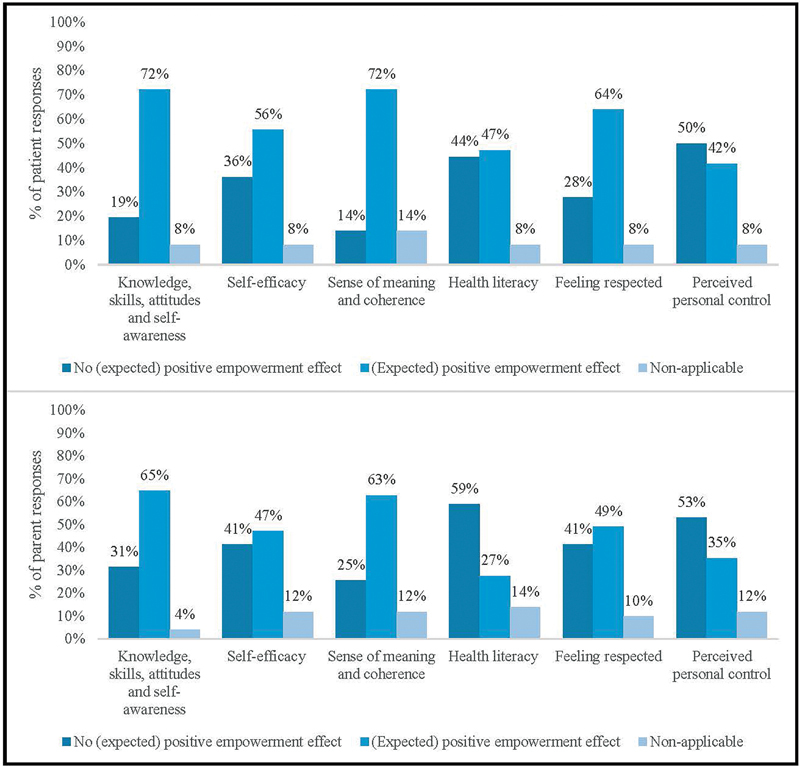
The percentage of (patient and parent) responses signaling an expected positive empowerment effect, relative to those responses not signaling such an effect – grouped into the components of patient empowerment model Bravo (2015). 35 Percentages are rounded to the nearest whole number. As a result, they may not add up to 100%.

Parents largely expected the website to have a positive effect on their empowerment, by generating the knowledge, skills, attitudes, and self-awareness necessary to influence their own behavior (65%) and providing a sense of meaning and coherence about their child's condition (63%). Parents did not expect the website to have as much of a positive effect on their feelings of respect (49%), self-efficacy (47%), perceived personal control (35%), and health literacy (27%). Notably, for perceived personal control and health literacy, responses signaling “no expected positive empowerment effect” (53% and 59%, respectively) outnumbered those signaling any “positive effect.” Displayed separately for patients and parents, Fig. 6 depicts the percentage of responses signaling an expected positive empowerment effect, relative to those responses not signaling such an effect.

### User Experiences

#### Healthcare Professionals Involved in the Care of ARM/HD

##### Website Content and Presentation

All healthcare professionals ( *n* = 20, 100%) found the website to be (at least reasonably) clear and comprehensive, intuitive and easy to navigate, with easy to find, accurate information and a clear target audience. Most respondents also found the website to be (at least reasonably) complete ( *n* = 18, 90%), with 14 considering it fully complete ( *n* = 14, 70%). The remaining two respondents were unsure (10%). Most respondents ( *n* = 18, 90%) were not aware of any additional resources that could be added to the website, with no specific suggestions provided. All respondents ( *n* = 20, 100%) found the website to have (at least a reasonably) reliable, professional appearance and appealing layout. Most respondents found the website to have (at least a reasonably) attractive font size and style ( *n* = 19, 95%), with (at least reasonably) informative images ( *n* = 19, 95%).

##### Website Inclusivity

Most healthcare professionals ( *n* = 17, 85%) indicated that they would find it helpful to have the website translated into their native language, with 11 additional languages proposed. The majority were unsure whether cultural considerations needed to be taken into account ( *n* = 12, 60%). Difficulties talking about sexuality in certain religious contexts was noted as an important consideration, with one respondent suggesting that native speakers check the wording of the website text.

##### Points for Improvement

Room for website improvement was detected by 35% of healthcare professionals ( *n* = 7), with a further 30% unsure ( *n* = 6). Points for improvement related to both website content/presentation and inclusivity. These included arranging the text differently (e.g., splitting content relevant to HD and ARM), improving the presentation of the website for patients (specifically adolescents), adding clear information on long-term problems, mentioning that there is the possibility of receiving help elsewhere in the case of insufficient support, and making translated links available.

##### Relevance to the Target Group

Most healthcare professionals found the website to be (at least reasonably) applicable to them ( *n* = 19, 95%) and would recommend it to colleagues ( *n* = 18, 90%) and other healthcare providers ( *n* = 15, 75%) with certainty, namely, stoma nurses, pediatricians, gynecologists, and urologists. Most respondents would also recommend the website to patients with ARM/HD ( *n* = 17, 85%) and their parents ( *n* = 16, 80%). Many ( *n* = 8, 40%) would recommend the website to someone else, namely, trainees, general practitioners, and other patient groups.

#### Patients with ARM/HD and Their Parents

##### Website Content and Presentation

Most patients and parents found the website to be (at least reasonably) clear and comprehensive ( *n* = 11, 92%; *n* = 14, 82%) and intuitive and easy to navigate ( *n* = 10, 83%; *n* = 15, 88%). Most patients and parents found it to have (at least reasonably) easy to find ( *n* = 9, 75%; *n* = 15, 88%), accurate ( *n* = 8, 67%; *n* = 11, 65%) information, with (at least a reasonably) clear target audience ( *n* = 11, 92%; *n* = 16, 94%). Notably, only one patient and one parent found the website to be fully complete, with six additional patients (50%) and seven parents (41%) responding “reasonably.” Most patients ( *n* = 8, 67%) and parents ( *n* = 15, 88%) were not aware of any additional resources that could be added to the website, with only one patient recommending Youthrally.org Facebook groups and one parent recommending an information booklet developed by a Dutch HD patient society. For most patients and parents, the website was found to have (at least a reasonably) reliable, professional appearance ( *n* = 11, 92%; *n* = 15, 88%, respectively), with (at least a reasonably) appealing layout ( *n* = 11, 92%; *n* = 14, 82%), attractive font size and style ( *n* = 11, 92%; *n* = 17, 100%), and informative images ( *n* = 9, 75%; *n* = 13, 76%).

##### Website Inclusivity

Half of the patients (50%) and most parents ( *n* = 16, 94%) would find it helpful to have the website translated into their native language, proposing five additional languages. Most patients ( *n* = 7, 58%) and parents ( *n* = 13, 76%) were unsure whether cultural considerations should be taken into account. The following considerations were explicitly noted: the importance of recognizing that a stoma bag is “not a bad thing,” LGBTQIA + inclusivity, multiculturalism (where in some cultures, sex is not talked about openly with parents), and the healthcare system (the culture of its professionals, lack of resources) that may hinder healthcare professionals' use and distribution of the website.

##### Points for Improvement

Room for website improvement was detected by 50% of patients ( *n* = 6) and 47% of parents ( *n* = 8), with a further 33% ( *n* = 4) and 41% ( *n* = 7) unsure. Points for improvement related to both website content/presentation and inclusivity. These included the need for more inclusive information (e.g., for LGBTQIA+ communities), additional information specific to target groups (e.g., for youngsters, adults, females, those with comorbidities such as Down's syndrome), recommendations from sexologists and physiotherapists, more concrete, in-depth/specific information that is less “redundant,” testimonials, information on how to communicate with a partner, information/links related to having children (providing clarity about the possible risk of them having the same condition), information on what a sexologist is and what their consultations would be like, and improved website design and navigation (“loosened up,” with more structure, vividness, pictures) and translations.

##### Relevance to the Target Group

Most patients ( *n* = 10, 83%) and parents ( *n* = 16, 94%) found the website to be (at least reasonably) applicable to them. Most patients would recommend the website to other patients with ARM/HD ( *n* = 8, 67%), parents ( *n* = 9, 75%), and healthcare providers ( *n* = 7, 58%) with certainty. Most parents reported that they too would recommend the website to other parents ( *n* = 11, 65%), patients ( *n* = 11, 65%) and healthcare providers ( *n* = 10, 59%). Many patients ( *n* = 6, 50%) and parents ( *n* = 6, 35%) would also recommend the website to third parties. For patients this included partners and trusted others and for parents, this included friends and caregivers in kindergarten or school.

Supplementary File 3 (available in the online version) provides an overview of survey questions and responses analyzed quantitatively.

## Discussion

The ERNICA/eUROGEN sexual support website was accessed and evaluated internationally by a diverse group of patients with ARM/HD, their parents, and healthcare professionals. Previous research clearly indicates that patients with ARM and HD have sexual support needs that are not addressed. 12 19 21 Our study further validates such needs, primarily for patients with ARM, with all those known to be sexually active found to experience sexual problems. The ERNICA/eUROGEN sexual support website is the first known tool designed to empower patients with ARM/HD, their parents, and healthcare professionals to address relevant sex-related topics in their interactions. This study reveals that for individuals living with/caring for those with these conditions, the website is considered an appropriate or relevant (“applicable”) tool that can be recommended to patients, parents, and healthcare providers, among others.

Healthcare professionals largely expected the website to have a positive effect on their structural empowerment, by providing information, support, and opportunities to learn and grow. A positive effect on their psychological empowerment was also expected, with the website thought to generate an increased sense of meaning, competence, self-determination, and a perceived ability to provide impact. Such positive expectations validate several barriers and empowerment needs used to inform initial development of the Dutch website, e.g., the need for information possibilities and knowledge about (possible) sexual problems. 22 However, despite aiming to address lack of time as a barrier to discussing sex-related topics with patients, 22 the website was not expected to empower healthcare professionals to “free up resources.” Even with access to the website, healthcare professionals may continue to encounter resource constraints in practice. This places individual empowerment within the context of a wider system, where constraints may be beyond individuals' direct scope of influence.

For both patients and parents, the website was also expected to generate a positive empowerment effect in several areas. Expectations were, however, lowest for self-efficacy, perceived personal control, and health literacy. These findings are not surprising, since the website was not developed with the intention of fostering an empowerment effect in these specific areas. 22 Even with access to the website, patients with ARM/HD and their parents may also not feel they have direct influence over whether sexual problems are “solved” or “improved,” especially in the case of anatomical differences. It is particularly notable that parents' empowerment needs were not studied, and therefore they were not used to inform development of the website. 22 If parents are to remain a key target group, exploring their barriers and empowerment needs will be key to maximizing the website's empowerment potential.

Although most patients and parents were not aware of any resources that could be added to the website, the majority did not find it fully complete, identifying several areas they considered to be missing. The website's inclusivity (e.g., of LGBTQIA+ communities) was also highlighted as an area for further attention. Ensuring the website reaches all members of its target group is key to maximizing its empowerment potential, as is the extent to which it (accurately) captures the diverse range of difficulties they experience. Moving forward, it will therefore be important to add/develop relevant content and resources and establish a system for continuous monitoring, maintenance, and evaluation. As European expertise networks, ERNICA and ERN eUROGEN are well placed to decide, together with stakeholder groups, on what content/resources should be added. Yet, with respondents proposing a number of additional target groups for the website, it will also be crucial for ERNICA and ERN eUROGEN to define its ongoing scope.

Exploring uptake of the tool and the factors influencing it was beyond the scope of this study. However, generating an empowerment effect relies, in part, on successful implementation of the website within the institutional context where patients, parents, and healthcare professionals interact. The interaction between these individuals is fundamental, with patients' sexual support needs addressed through the empowerment of all parties involved. In this study, the Kanter, 33 Thomas and Velthouse, 34 and Bravo 35 models are used to conceptualize empowerment. Altogether, these models form a health professional–patient empowerment model, 22 based on the theory that empowered healthcare professionals will empower patients (and their parents) to address sexual support needs, ultimately resulting in improved patient outcomes. 22 36 Notably, recent research has indicated a relationship between professional empowerment and the implementation of evidence-based practice. 37 Website translations are recommended to increase the tool's accessibility for patients, parents, and healthcare professionals internationally. Yet, the tool may be limited in the extent to which it can address institutional challenges. Implementation research 38 may be a valuable next step to explore uptake of the website in institutional contexts, specifically focused on identifying barriers and facilitators and testing effective strategies. Such research may also provide insight into (context-specific) cultural factors and religious values warranting consideration.

### Strengths and Limitations

Although a strength of this study is its theoretical underpinning, the empowerment models applied were not developed for use in the context of sexual support needs. Expected empowerment effects were also captured using self-reported survey questions not assessed for their (cross-cultural) psychometric robustness. Although survey questions were based on those employed by Petit-Steeghs in the previous evaluation of the Dutch website, 22 they were not specifically pre-tested for feasibility and understandability. Furthermore, while absolute and relative values were calculated for each statement included in Part B of the surveys (see Supplementary File 3 (available in the online version)), the expected “empowerment effect” was only calculated at the level of the relevant empowerment component.

Having the evaluation survey available in 24 different languages increases the representativeness of this study, with a number of respondents choosing to respond in their native language. However, the survey (and its results where relevant) were translated using machine translation, risking misinterpretation. Furthermore, although the survey was available in multiple languages, the website itself was available in Dutch and English only. We will have missed non-Dutch/English speakers, as well as those lacking the necessary digital resources and skills. Website translations should be pursued moving forward.

The study's generalizability is limited by its relatively small sample size, which may have been influenced by the timing of dissemination (over summer) and/or the taboo often associated with the subject matter. Furthermore, most healthcare professional respondents were pediatric surgeons and only one patient with HD responded. No sexologist, psychologist, pelvic floor specialist, or gynecologist took part.

This study serves as an initial evaluation of the ERNICA/eUROGEN sexual support website. Repeated evaluations, with larger samples, are warranted in the future. Larger samples will also allow for more advanced analyses and sub-group comparisons. Although our study measured the expected empowerment effect only, it has provided insight into factors warranting further exploration through dedicated implementation research. A longitudinal study design may be considered for the future, to capture changes in stakeholder behavior over time. ERN hospitals and ARM/HD patient organizations are well placed to support these efforts.

## Conclusion

The ERNICA/eUROGEN sexual support website shows promise in empowering patients with ARM/HD, their parents, and healthcare professionals by fostering learning and offering relevant information and support. To increase the website's empowerment potential, attention should be paid to the website's inclusivity (e.g., of LGBTQIA+ individuals), its cultural sensitivity and accessibility. ERNICA and ERN eUROGEN are well placed to define the website's ongoing scope, drive the development of (translated) content/resources, and establish a system for continuous monitoring, maintenance, and evaluation. Although the website has the potential to support healthcare professionals' empowerment, institutional barriers may persist. Implementation research is recommended to support uptake of the tool within the (institutional) contexts where patients (HD/ARM), parents, and healthcare professionals interact.
