# Supplementary material for: Evaluating the Empowerment Potential of an International Sexual Support Website for Patients with Anorectal Malformations and Hirschsprung Disease, their Parents and Healthcare Providers
Source: Eur J Pediatr Surg. 2025 Jul 9;35(6):494–504. doi: 10.1055/a-2635-7802 (PMC12611478; doi:10.1055/a-2635-7802)
Supplement: Supplementary file 1 — Supplementary Material [file 10-1055-a-2635-7802-s2024097093oa.pdf]

Supplementary File 1

Overview of Part B statements aligned to their relevant empowerment component

Healthcare Professionals

#	Statement	Component based on the models of Kanter[a] and Thomas and Velthouse[b]
1.	The website increases my knowledge about possible sexual problems for these patient groups.	Structural—Information
2.	The website increases my knowledge about possibilities to support patients with sexual problems.	Structural—Information
3.	The website increases my knowledge about how the topic of sexuality can be included as a standard part of the care process.	Structural—Information
4.	The website can provide me with support when discussing the topic of sexuality.	Structural—Support
5.	The website can support me to support patients to improve their sexual problems.	Structural—Support
6.	The website can support me to integrate the topic of sexuality in current work processes.	Structural—Support
7.	The website can help me to free up time to broaden my skills and knowledge to support patients with sexual problems.	Structural—Resources
8.	The website helps me to free up financial resources to broaden my skills and knowledge to support patients with sexual problems.	Structural—Resources
9.	The website helps me to free up time to support patients with sexual problems.	Structural—Resources
10.	The website offers possibilities to acquire knowledge and skills about the sexual problems experienced by these patient groups.	Structural—Opportunities
11.	The website offers possibilities to support the patient groups to improve their sexual health.	Structural—Opportunities
12.	The website offers possibilities to integrate the topic of sexuality as a standard part of the care process.	Structural—Opportunities
13.	The website provides insight into the importance of improving the sexual health of the patient groups.	Psychological—Meaningfulness
14.	The website provides insight into the importance of improving patient support for sexual problems.	Psychological—Meaningfulness
15.	The website makes me feel more responsible for improving support for patient groups with sexual problems.	Psychological—Meaningfulness
16.	The website makes me feel better able to introduce the subject of sexuality during a consultation.	Psychological—Competence
17.	The website makes me feel better able to discuss the subject of sexuality with patients.	Psychological—Competence
18.	The website makes me feel better able to improve support for patients with their sexual problems.	Psychological—Competence
19.	The website offers me the possibility to introduce the topic of sexuality during consultations.	Psychological—Self-determination
20.	The website helps me to introduce the topic of sexuality during patient consultations.	Psychological—Self-determination
21.	The website helps me to introduce the topic of sexuality among colleagues.	Psychological—Self-determination
22.	As a result of the website, I know better how I can raise the topic of sexuality in patient consultations.	Psychological—Impact
23.	As a result of the website, I know better how to integrate the subject of sexuality in current work processes.	Psychological—Impact
24.	As a result of the website, I know better how I can ensure that more time and resources can be made available to pay attention to the subject of sexuality.	Psychological—Impact

 © 2025. The Author(s).

Patients

#	Statement	Component based on Bravo model[c]
1.	The information on the website increases my knowledge about possible sexual problems related to the condition.	Knowledge, skills, attitudes, and self-awareness necessary to influence their own health behavior
2.	The information on the website provides me with skills to deal with possible sexual problems related to the condition.	Knowledge, skills, attitudes, and self-awareness necessary to influence their own health behavior
3.	The information on the website makes me more aware of the sexual problems involved with the condition.	Knowledge, skills, attitudes, and self-awareness necessary to influence their own health behavior
4.	Thanks to the information on the website, I feel confident that I can solve possible (future) sexual problems.	Self-efficacy
5.	Thanks to the information on the website, I feel confident that I can improve my sexual health.	Self-efficacy
6.	Thanks to the information on the website, I feel confident that I can arrange support for possible (future) sexual problems.	Self-efficacy
7.	Thanks to the information on the website, I see the importance of being actively involved with my sexual health.	Sense of meaning and coherence about their condition
8.	Thanks to the information on the website, I realize that a healthy sexual life can affect how I feel.	Sense of meaning and coherence about their condition
9.	Thanks to the information on the website, I am aware that my sexual health forms part of my quality of life.	Sense of meaning and coherence about their condition
10.	The information on the website is sufficient to be able to solve possible (future) sexual problems.	Health literacy
11.	The information on the website is sufficient to improve my sexual health.	Health literacy
12.	The information on the website is sufficient to be able to arrange professional support for (future) sexual problems.	Health literacy
13.	Thanks to the information on the website, I feel more understood as a patient.	Feeling respected
14.	Thanks to the information on the website, I feel more supported as a patient.	Feeling respected
15.	Thanks to the information on the website, I feel like healthcare providers are interested in my sexual health.	Feeling respected
16.	Thanks to the information on the website, I think I have the knowledge and skills to solve possible (future) sexual problems.	Perceived personal control over health and healthcare
17.	The information on the website makes me feel able to improve my sexual health.	Perceived personal control over health and healthcare
18.	Because of the information on the website, I feel able to solve possible sexual problems.	Perceived personal control over health and healthcare

 © 2025. The Author(s).

Parents

#	Statement	Component based on Bravo model[c]
1.	The information on the website increases my knowledge about possible sexual problems related to the condition.	Knowledge, skills, attitudes, and self-awareness necessary to influence their own health behavior
2.	The information on the website provides me with skills to support my child with possible (future) sexual problems related to the condition.	Knowledge, skills, attitudes, and self-awareness necessary to influence their own health behavior
3.	The information on the website makes me more aware of the sexual problems involved with the condition.	Knowledge, skills, attitudes, and self-awareness necessary to influence their own health behavior
4.	Thanks to the information on the website, I feel confident to help my child solve possible (future) sexual problems.	Self-efficacy
5.	Thanks to the information on the website, I feel confident to help my child improve his/her sexual health.	Self-efficacy
6.	Thanks to the information on the website, I feel confident to arrange professional support for possible (future) sexual problems experienced by my child.	Self-efficacy
7.	Thanks to the information on the website, I see the importance of being consciously engaged in my child's sexual health.	Sense of meaning and coherence about their condition
8.	Thanks to the information on the website, I realize that a healthy sexual life can affect how you feel.	Sense of meaning and coherence about their condition
9.	Thanks to the information on the website, I am aware that my child's sexual health forms part of their physical and psychological well-being.	Sense of meaning and coherence about their condition
10.	The information on the website is sufficient to support my child to solve possible (future) sexual problems.	Health literacy
11.	The information on the website is sufficient to support my child to improve their sexual health.	Health literacy
12.	The information on the website is sufficient to be able to arrange professional support for my child for possible (future) sexual problems.	Health literacy
13.	Thanks to the information on the website, I feel more understood as a parent of a child with Hirschsprung disease/anorectal malformation.	Feeling respected
14.	Thanks to the information on the website, I feel more supported as a parent of a child with Hirschsprung disease/anorectal malformation.	Feeling respected
15.	Thanks to the information on the website, I feel like healthcare providers are interested in my child's sexual health.	Feeling respected
16.	Thanks to the information on the website, I think I have the knowledge and skills to support my child to solve possible (future) sexual problems.	Perceived personal control over health and healthcare
17.	Thanks to information on the website, I feel able to support my child to improve their sexual health.	Perceived personal control over health and healthcare
18.	Thanks to information on the website, I feel able to help my child solve (future) sexual problems.	Perceived personal control over health and healthcare

Notes: [a]Kanter RM. Men and women of the corporation revisited: interview with Rosabeth Moss Kanter. Human Resource Management 1987;26 (2):257–263.
[b]Thomas KW, Velthouse BA. Cognitive elements of empowerment: an "interpretive" model of intrinsic task motivation. Acad Manage Rev 1990;15 (4):666–681.
[c]Bravo P, Edwards A, Barr PJ, et al. Conceptualising patient empowerment: a mixed methods study. BMC Health Serv Res 2015;15:252.

 © 2025. The Author(s).

Supplementary File 2

Available Survey Languages

1. English (EN)
2. Bulgarian (BG)
3. Czech (CS)
4. Danish (DA)
5. German (DE)
6. Greek (EL)
7. Spanish (ES)
8. Estonian (ET)
9. Finnish (FI)
10. French (FR)
11. Irish (GA)
12. Hungarian (HU)
13. Croatian (HR)
14. Italian (IT)
15. Lithuanian (LT)
16. Latvian (LV)
17. Maltese (MT)
18. Dutch (NL)
19. Portugese (PT)
20. Polish (PL)
21. Romanian (RO)
22. Swedish (SV)
23. Slovenian (SL)
24. Slovak (SK)

 © 2025. The Author(s).

Supplementary File 3

Survey questions and quantitatively analyzed survey responses

Healthcare professionals involved in the care of ARM/HD			
PART A		*n*	%[a]
**What is your position?**	Pediatric surgeon	14	70
	Pediatrician	2	10
	Nurse	1	5
	Sexologist	0	0
	Psychologist	0	0
	Pelvic floor specialist	0	0
	Urologist	2	10
	Gynecologist	0	0
	Pediatric surgeon-urologist	1	5
	Other (Please specify)	0	0
**Do you work in a hospital-based setting?**	Yes	20	100
	No	0	0
	**[If yes] What is the name of your hospital? (Optional)**	16	80
	Ospedale papa Giovanni XXIII Bergamo	1	6
	Oslo University Hospital	1	6
	Sant Joan de Déu Barcelona Children's Hospital	3	19
	Odense University Hospital	1	6
	AO SS Antonio e Biagio e Cesare Arrigo	1	6
	Lund University Hospital	2	13
	University Hospital Motol	1	6
	Lithuanian University of Health Sciences Kaunas Clinics	1	6
	Fattouma Bourguiba Hospital	1	6
	University Clinic Tuebingen	1	6
	Radboud University Medical Centre	1	6
	University Hospital Mannheim	1	6
	New Children's Hospital, University of Helsinki	1	6
	**[If no] What is your work base?**	0	0
**What is your biological sex?**	Male	6	30
	Female	14	70
	Don't wish to state	0	0
**What age category do you fit into?**	<30	3	15
	31–50	9	45
	>50	8	40
PART B		*n*	%
**The website increases my knowledge about possible sexual problems for these patient groups.**	Strongly disagree	0	0
	Disagree	0	0
	Neutral	2	10
	Agree	11	55
	Strongly agree	7	35
	Non-applicable	0	0

 © 2025. The Author(s).

(*Continued*)

Healthcare professionals involved in the care of ARM/HD			
**The website increases my knowledge about possibilities to support patients with sexual problems.**	Strongly disagree	0	0
	Disagree	1	5
	Neutral	1	5
	Agree	11	55
	Strongly agree	7	35
	Non-applicable	0	0
**The website increases my knowledge about how the topic of sexuality can be included as a standard part of the care process.**	Strongly disagree	0	0
	Disagree	0	0
	Neutral	1	5
	Agree	14	70
	Strongly agree	5	25
	Non-applicable	0	0
**The website can provide me with support when discussing the topic of sexuality.**	Strongly disagree	0	0
	Disagree	0	0
	Neutral	2	10
	Agree	9	45
	Strongly agree	9	45
	Non-applicable	0	0
**The website can support me to support patients to improve their sexual problems.**	Strongly disagree	0	0
	Disagree	0	0
	Neutral	2	10
	Agree	13	65
	Strongly agree	5	25
	Non-applicable	0	0
**The website can support me to integrate the topic of sexuality in current work processes.**	Strongly disagree	0	0
	Disagree	0	0
	Neutral	1	5
	Agree	12	60
	Strongly agree	7	35
	Non-applicable	0	0
**The website can help me to free up time to broaden my skills and knowledge to support patients with sexual problems.**	Strongly disagree	0	0
	Disagree	2	10
	Neutral	8	40
	Agree	8	40
	Strongly agree	2	10
	Non-applicable	0	0
**The website helps me to free up financial resources to broaden my skills and knowledge to support patients with sexual problems.**	Strongly disagree	1	5
	Disagree	5	25
	Neutral	12	60
	Agree	2	10
	Strongly agree	0	0
	Non-applicable	0	0

(*Continued*)

(*Continued*)

Healthcare professionals involved in the care of ARM/HD			
**The website helps me to free up time to support patients with sexual problems.**	Strongly disagree	0	0
	Disagree	5	25
	Neutral	4	20
	Agree	9	45
	Strongly agree	2	10
	Non-applicable	0	0
**The website offers possibilities to acquire knowledge and skills about the sexual problems experienced by these patient groups.**	Strongly disagree	0	0
	Disagree	0	0
	Neutral	3	15
	Agree	9	45
	Strongly agree	8	40
	Non-applicable	0	0
**The website offers possibilities to support the patient groups to improve their sexual health.**	Strongly disagree	0	0
	Disagree	1	5
	Neutral	1	5
	Agree	12	60
	Strongly agree	6	30
	Non-applicable	0	0
**The website offers possibilities to integrate the topic of sexuality as a standard part of the care process.**	Strongly disagree	0	0
	Disagree	0	0
	Neutral	4	20
	Agree	6	30
	Strongly agree	10	50
	Non-applicable	0	0
**The website provides insight into the importance of improving the sexual health of the patient groups.**	Strongly disagree	0	0
	Disagree	0	0
	Neutral	0	0
	Agree	10	50
	Strongly agree	10	50
	Non-applicable	0	0
**The website provides insight into the importance of improving patient support for sexual problems.**	Strongly disagree	0	0
	Disagree	0	0
	Neutral	1	5
	Agree	11	55
	Strongly agree	8	40
	Non-applicable	0	0
**The website makes me feel more responsible for improving support for patient groups with sexual problems.**	Strongly disagree	0	0
	Disagree	2	10
	Neutral	6	30
	Agree	4	20
	Strongly agree	8	40
	Non-applicable	0	0

 © 2025. The Author(s).

(*Continued*)

Healthcare professionals involved in the care of ARM/HD			
**The website makes me feel better able to introduce the subject of sexuality during a consultation.**	Strongly disagree	0	0
	Disagree	3	15
	Neutral	1	5
	Agree	9	45
	Strongly agree	7	35
	Non-applicable	0	0
**The website makes me feel better able to discuss the subject of sexuality with patients.**	Strongly disagree	0	0
	Disagree	3	15
	Neutral	1	5
	Agree	10	50
	Strongly agree	6	30
	Non-applicable	0	0
**The website makes me feel better able to improve support for patients with their sexual problems.**	Strongly disagree	0	0
	Disagree	1	5
	Neutral	6	30
	Agree	6	30
	Strongly agree	7	35
	Non-applicable	0	0
**The website offers me the possibility to introduce the topic of sexuality during consultations.**	Strongly disagree	0	0
	Disagree	2	10
	Neutral	1	5
	Agree	9	45
	Strongly agree	8	40
	Non-applicable	0	0
**The website helps me to introduce the topic of sexuality during patient consultations.**	Strongly disagree	0	0
	Disagree	1	5
	Neutral	1	5
	Agree	11	55
	Strongly agree	7	35
	Non-applicable	0	0
**The website helps me to introduce the topic of sexuality among colleagues.**	Strongly disagree	0	0
	Disagree	1	5
	Neutral	7	35
	Agree	4	20
	Strongly agree	8	40
	Non-applicable	0	0
**As a result of the website, I know better how I can raise the topic of sexuality in patient consultations.**	Strongly disagree	0	0
	Disagree	1	5
	Neutral	3	15
	Agree	10	50
	Strongly agree	6	30
	Non-applicable	0	0

(*Continued*)

 © 2025. The Author(s).

(*Continued*)

Healthcare professionals involved in the care of ARM/HD			
**As a result of the website, I know better how to integrate the subject of sexuality in current work processes.**	Strongly disagree	0	0
	Disagree	1	5
	Neutral	2	10
	Agree	13	65
	Strongly agree	4	20
	Non-applicable	0	0
**As a result of the website, I know better how I can ensure that more time and resources can be made available to pay attention to the subject of sexuality.**	Strongly disagree	0	0
	Disagree	4	20
	Neutral	4	20
	Agree	6	30
	Strongly agree	6	30
	Non-applicable	0	0
PART C		***n***	**%**
**Is the information on the website presented clearly and comprehensibly?**	Yes	18	90
	Reasonably	2	10
	No	0	0
	I don't know	0	0
**Is the information on the website accurate?**	Yes	18	90
	Reasonably	2	10
	No	0	0
	I don't know	0	0
**Is the information on the website complete?**	Yes	14	70
	Reasonably	4	20
	No	0	0
	I don't know	2	10
**Are you aware of any additional resources that may be included on the website?**	Yes	2	10
	No	18	90
**Is the information on the website easy to find?**	Yes	16	80
	Reasonably	4	20
	No	0	0
	I don't know	0	0
**Is it clear who the website is intended for?**	Yes	17	85
	Reasonably	3	15
	No	0	0
	I don't know	0	0
**Is the layout appealing?**	Yes	13	65
	Reasonably	7	35
	No	0	0
	I don't know	0	0
**Are the images informative?**	Yes	14	70
	Reasonably	5	25
	No	0	0
	I don't know	1	5

 © 2025. The Author(s).

(*Continued*)

Healthcare professionals involved in the care of ARM/HD			
**Is the font size and style on the website attractive to you?**	Yes	13	65
	Reasonably	6	30
	No	1	5
	I don't know	0	0
**Does the website have a reliable/ professional appearance?**	Yes	14	70
	Reasonably	6	30
	No	0	0
	I don't know	0	0
**Is the website intuitive and easy to navigate?**	Yes	18	90
	Reasonably	2	10
	No	0	0
	I don't know	0	0
**Is the website applicable for you? (See** www.dictionary.com/browse/applicable**)**	Yes	16	80
	Reasonably	3	15
	No	0	0
	I don't know	1	5
**Would you recommend the website to patients with an anorectal malformation or Hirschsprung disease?**	Yes	17	85
	Probably	2	10
	Maybe	1	5
	No	0	0
	I don't know	0	0
**Would you recommend the website to parents of children with an anorectal malformation or Hirschsprung disease?**	Yes	16	80
	Probably	3	15
	Maybe	1	5
	No	0	0
	I don't know	0	0
**Would you recommend the website to colleagues?**	Yes	18	90
	Probably	1	5
	Maybe	1	5
	No	0	0
	I don't know	0	0
**Would you recommend the website to other healthcare providers?**	Yes	15	75
	Probably	4	20
	Maybe	1	5
	No	0	0
	I don't know	0	0
**Would you recommend the website to someone else?**	Yes	8	40
	Probably	3	15
	Maybe	4	20
	No	1	5
	I don't know	4	20
**Do you think anything needs to be improved on the website?**	Yes	7	35
	No	7	35
	I don't know	6	30

(*Continued*)

 © 2025. The Author(s).

(*Continued*)

Healthcare professionals involved in the care of ARM/HD			
**Do you think that it would be helpful to have the website translated into your native language?**	Yes	17	85
	No	2	10
	I don't know	1	5
**Are there any cultural considerations that should be taken into account for the website?**	Yes	3	15
	No	5	25
	I don't know	12	60

[a]Percentages are rounded to the nearest whole number. As a result, they may not add up to 100%.

Patients with ARM/HD			
PART A		*n*	%[a]
**Which condition have you been diagnosed with?**	Anorectal malformation	11	92
	Hirschsprung disease	1	8
**How old are you?**	20–25 years	2	17
	26–30 years	4	33
	31–40 years	4	33
	41–50 years	0	0
	50+ years	2	17
**Which country do you live in?**	Germany	5	42
	The United Kingdom	4	33
	The Netherlands	1	8
	Australia	1	8
	The United States of America	1	8
**Which country were you born in?**	Germany	6	50
	The Netherlands	1	8
	The United Kingdom	3	25
	Ireland	1	8
	The United States of America	1	8
**Did you grow up in a rural or urban environment?**	Rural	6	50
	Urban	4	33
	Both	2	17
**What is your biological sex?**	Male	5	42
	Female	7	58
	Don't wish to say	0	0
**Are you currently receiving medical care for your condition?**	Yes	7	58
	No	5	42
**Do you experience bowel irrigations?**	Yes	4	33
	No	8	67
**Have you experienced bowel irrigations in the past?**	Yes	8	67
	No	4	33
**Do you have a stoma? (Opening in the belly wall for waste to pass through)**	Yes	6	50
	No	6	50

 © 2025. The Author(s).

(*Continued*)

Patients with ARM/HD			
**Have you been/are you currently sexually active?**	Yes	11	92
	No	1	8
	**[If yes] To what extent have you experienced sexual problems?**		
	No, I have not experienced any sexual problems.	0	0
	Yes, I have experienced sexual problems in the past.	7	64
	Yes, I am currently experiencing sexual problems.	0	0
	Yes, I experience sexual problems both now and in the past.	4	36
PART B		***n***	%
**The information on the website increases my knowledge about possible sexual problems related to the condition.**	Strongly disagree	0	0
	Disagree	1	8
	Neutral	1	8
	Agree	6	50
	Strongly agree	3	25
	Non-applicable	1	8
**The information on the website provides me with skills to deal with possible sexual problems related to the condition.**	Strongly disagree	0	0
	Disagree	0	0
	Neutral	4	33
	Agree	3	25
	Strongly agree	4	33
	Non-applicable	1	8
**The information on the website makes me more aware of the sexual problems involved with the condition.**	Strongly disagree	0	0
	Disagree	0	0
	Neutral	1	8
	Agree	6	50
	Strongly agree	4	33
	Non-applicable	1	8
**Thanks to the information on the website, I feel confident that I can solve possible (future) sexual problems.**	Strongly disagree	0	0
	Disagree	1	8
	Neutral	5	42
	Agree	2	17
	Strongly agree	3	25
	Non-applicable	1	8
**Thanks to the information on the website, I feel confident that I can improve my sexual health.**	Strongly disagree	0	0
	Disagree	0	0
	Neutral	5	42
	Agree	3	25
	Strongly agree	3	25
	Non-applicable	1	8
**Thanks to the information on the website, I feel confident that I can arrange support for possible (future) sexual problems.**	Strongly disagree	0	0
	Disagree	1	8
	Neutral	1	8
	Agree	5	42
	Strongly agree	4	33
	Non-applicable	1	8

(*Continued*)

 © 2025. The Author(s).

(*Continued*)

Patients with ARM/HD			
**Thanks to the information on the website, I see the importance of being actively involved with my sexual health.**	Strongly disagree	0	0
	Disagree	0	0
	Neutral	1	8
	Agree	5	42
	Strongly agree	5	42
	Non-applicable	1	8
**Thanks to the information on the website, I realize that a healthy sexual life can affect how I feel.**	Strongly disagree	0	0
	Disagree	0	0
	Neutral	3	25
	Agree	4	33
	Strongly agree	3	25
	Non-applicable	2	17
**Thanks to the information on the website, I am aware that my sexual health forms part of my quality of life.**	Strongly disagree	0	0
	Disagree	0	0
	Neutral	1	8
	Agree	5	42
	Strongly agree	4	33
	Non-applicable	2	17
**The information on the website is sufficient to be able to solve possible (future) sexual problems.**	Strongly disagree	0	0
	Disagree	2	17
	Neutral	3	25
	Agree	5	42
	Strongly agree	1	8
	Non-applicable	1	8
**The information on the website is sufficient to improve my sexual health.**	Strongly disagree	1	8
	Disagree	1	8
	Neutral	4	33
	Agree	4	33
	Strongly agree	1	8
	Non-applicable	1	8
**The information on the website is sufficient to be able to arrange professional support for (future) sexual problems.**	Strongly disagree	1	8
	Disagree	0	0
	Neutral	4	33
	Agree	5	42
	Strongly agree	1	8
	Non-applicable	1	8
**Thanks to the information on the website, I feel more understood as a patient.**	Strongly disagree	0	0
	Disagree	1	8
	Neutral	1	8
	Agree	5	42
	Strongly agree	4	33
	Non-applicable	1	8

(*Continued*)

Patients with ARM/HD			
**Thanks to the information on the website, I feel more supported as a patient.**	Strongly disagree	1	8
	Disagree	0	0
	Neutral	2	17
	Agree	4	33
	Strongly agree	4	33
	Non-applicable	1	8
**Thanks to the information on the website, I feel like healthcare providers are interested in my sexual health.**	Strongly disagree	1	8
	Disagree	1	8
	Neutral	3	25
	Agree	3	25
	Strongly agree	3	25
	Non-applicable	1	8
**Thanks to the information on the website, I think I have the knowledge and skills to solve possible (future) sexual problems.**	Strongly disagree	0	0
	Disagree	1	8
	Neutral	5	42
	Agree	3	25
	Strongly agree	2	17
	Non-applicable	1	8
**The information on the website makes me feel able to improve my sexual health.**	Strongly disagree	1	8
	Disagree	1	8
	Neutral	3	25
	Agree	4	33
	Strongly agree	2	17
	Non-applicable	1	8
**Because of the information on the website, I feel able to solve possible sexual problems.**	Strongly disagree	1	8
	Disagree	1	8
	Neutral	5	42
	Agree	2	17
	Strongly agree	2	17
	Non-applicable	1	8
PART C		**n**	**%**
**Is the information on the website presented clearly and comprehensibly?**	Yes	9	75
	Reasonably	2	17
	No	0	0
	I don't know	1	8
**Is the information on the website accurate?**	Yes	7	58
	Reasonably	1	8
	No	1	8
	I don't know	3	25
**Is the information on the website complete?**	Yes	1	8
	Reasonably	6	50
	No	3	25
	I don't know	2	17

(*Continued*)

 © 2025. The Author(s).

(*Continued*)

Patients with ARM/HD			
**Are you aware of any additional resources that may be included on the website?**	Yes	4	33
	No	8	67
**Is the information on the website easy to find?**	Yes	9	75
	Reasonably	0	0
	No	2	17
	I don't know	1	8
**Is it clear who the website is intended for?**	Yes	11	92
	Reasonably	0	0
	No	0	0
	I don't know	1	8
**Is the layout appealing?**	Yes	6	50
	Reasonably	5	42
	No	0	0
	I don't know	1	8
**Are the images informative?**	Yes	6	50
	Reasonably	3	25
	No	0	0
	I don't know	3	25
**Is the font size and style on the website attractive to you?**	Yes	7	58
	Reasonably	4	33
	No	0	0
	I don't know	1	8
**Does the website have a reliable/professional appearance?**	Yes	9	75
	Reasonably	2	17
	No	0	0
	I don't know	1	8
**Is the website intuitive and easy to navigate?**	Yes	10	83
	Reasonably	0	0
	No	1	8
	I don't know	1	8
**Is the website applicable for you? (See www.dictionary.com/browse/applicable)**	Yes	7	58
	Reasonably	3	25
	No	1	8
	I don't know	1	8
**Would you recommend the website to patients with an anorectal malformation or Hirschsprung disease?**	Yes	8	67
	Probably	2	17
	Maybe	1	8
	No	0	0
	I don't know	1	8

 © 2025. The Author(s).

(*Continued*)

Patients with ARM/HD			
**Would you recommend the website to parents of children with an anorectal malformation or Hirschsprung disease?**	Yes	9	75
	Probably	0	0
	Maybe	2	17
	No	0	0
	I don't know	1	8
**Would you recommend the website to healthcare providers?**	Yes	7	58
	Probably	2	17
	Maybe	2	17
	No	0	0
	I don't know	1	8
**Would you recommend the website to someone else?**	Yes	6	50
	Probably	2	17
	Maybe	3	25
	No	0	0
	I don't know	1	8
**Do you think anything needs to be improved on the website?**	Yes	6	50
	No	2	17
	I don't know	4	33
**Do you think that it would be helpful to have the website translated into your native language?**	Yes	6	50
	No	3	25
	I don't know	3	25
**Are there any cultural considerations that should be taken into account for the website?**	Yes	2	17
	No	3	25
	I don't know	7	58

[a]Percentages are rounded to the nearest whole number. As a result, they may not add up to 100%.

 © 2025. The Author(s).

Parents of patients with ARM/HD			
PART A		*n*	%[a]
**Which condition has your child been diagnosed with?**	Anorectal malformation	9	53
	Hirschsprung disease	8	47
**How old is your child?**	≤5 years	2	12
	6–11 years	6	35
	11–15 years	3	18
	15–20 years	2	12
	≥21 years	4	24
**Which country does your child live in?**	Germany	10	59
	The Netherlands	3	18
	Sweden	1	6
	France	2	12
	The United Kingdom	1	6
**Which country was your child born in?**	Germany	10	59
	The Netherlands	3	18
	Sweden	1	6
	France	2	12
	The United Kingdom	1	6
**Did they grow up in a rural or urban environment?**	Rural	4	24
	Urban	10	59
	Both	3	18
**What is their biological sex?**	Male	9	53
	Female	8	47
	Don't wish to say	0	0
**Is your child currently receiving medical care for their condition?**	Yes	14	82
	No	3	18
	I don't know	0	0
**Does your child experience bowel irrigations?**	Yes	8	47
	No	9	53
	I don't know	0	0
**Has your child experienced bowel irrigations in the past?**	Yes	10	59
	No	7	41
	I don't know	0	0
**Does your child have a stoma? (Opening in the belly wall for waste to pass through)**	Yes	1	6
	No	16	94
	I don't know	0	0
**Has your child been/are they currently sexually active?**	Yes	2	12
	No	14	82
	I don't know	1	6
	**[If yes] To what extent has your child experienced sexual problems?**		
	No, my child has not experienced any sexual problems.	0	0
	Yes, my child has experienced sexual problems in the past.	1	50
	Yes, my child is currently experiencing sexual problems.	0	0
	Yes, my child experiences current and past sexual problems	0	0
	I don't know	1	50
	My child is too young to experience sexual problems.	0	0

 © 2025. The Author(s).

(*Continued*)

Parents of patients with ARM/HD			
PART B		*n*	%
**The information on the website increases my knowledge about possible sexual problems related to the condition.**	Strongly disagree	0	0
	Disagree	3	18
	Neutral	2	12
	Agree	7	41
	Strongly agree	4	24
	Non-applicable	1	6
**The information on the website provides me with skills to support my child with possible (future) sexual problems related to the condition.**	Strongly disagree	1	6
	Disagree	2	12
	Neutral	3	18
	Agree	7	41
	Strongly agree	4	24
	Non-applicable	0	0
**The information on the website makes me more aware of the sexual problems involved with the condition.**	Strongly disagree	0	0
	Disagree	0	0
	Neutral	5	29
	Agree	7	41
	Strongly agree	4	24
	Non-applicable	1	6
**Thanks to the information on the website, I feel confident to help my child solve possible (future) sexual problems.**	Strongly disagree	1	6
	Disagree	4	24
	Neutral	2	12
	Agree	4	24
	Strongly agree	5	29
	Non-applicable	1	6
**Thanks to the information on the website, I feel confident to help my child improve his/her sexual health.**	Strongly disagree	1	6
	Disagree	3	18
	Neutral	3	18
	Agree	4	24
	Strongly agree	3	18
	Non-applicable	3	18
**Thanks to the information on the website, I feel confident to arrange professional support for possible (future) sexual problems experienced by my child.**	Strongly disagree	2	12
	Disagree	1	6
	Neutral	4	24
	Agree	5	29
	Strongly agree	3	18
	Non-applicable	2	12
**Thanks to the information on the website, I see the importance of being consciously engaged in my child's sexual health.**	Strongly disagree	0	0
	Disagree	0	0
	Neutral	4	24
	Agree	4	24
	Strongly agree	6	35
	Non-applicable	3	18

(*Continued*)

 © 2025. The Author(s).

(*Continued*)

Parents of patients with ARM/HD			
**Thanks to the information on the website, I realize that a healthy sexual life can affect how you feel.**	Strongly disagree	0	0
	Disagree	1	6
	Neutral	4	24
	Agree	4	24
	Strongly agree	7	41
	Non-applicable	1	6
**Thanks to the information on the website, I am aware that my child's sexual health forms part of their physical and psychological well-being.**	Strongly disagree	0	0
	Disagree	0	0
	Neutral	4	24
	Agree	3	18
	Strongly agree	8	47
	Non-applicable	2	12
**The information on the website is sufficient to support my child to solve possible (future) sexual problems.**	Strongly disagree	4	24
	Disagree	2	12
	Neutral	4	24
	Agree	3	18
	Strongly agree	2	12
	Non-applicable	2	12
**The information on the website is sufficient to support my child to improve their sexual health.**	Strongly disagree	1	6
	Disagree	4	24
	Neutral	5	29
	Agree	3	18
	Strongly agree	2	12
	Non-applicable	2	12
**The information on the website is sufficient to be able to arrange professional support for my child for possible (future) sexual problems.**	Strongly disagree	2	12
	Disagree	1	6
	Neutral	7	41
	Agree	2	12
	Strongly agree	2	12
	Non-applicable	3	18
**Thanks to the information on the website, I feel more understood as a parent of a child with Hirschsprung disease/ anorectal malformation.**	Strongly disagree	1	6
	Disagree	2	12
	Neutral	2	12
	Agree	5	29
	Strongly agree	6	35
	Non-applicable	1	6
**Thanks to the information on the website, I feel more supported as a parent of a child with Hirschsprung disease/ anorectal malformation.**	Strongly disagree	1	6
	Disagree	2	12
	Neutral	3	18
	Agree	4	24
	Strongly agree	6	35
	Non-applicable	1	6

 © 2025. The Author(s).

(*Continued*)

Parents of patients with ARM/HD			
**Thanks to the information on the website, I feel like healthcare providers are interested in my child's sexual health.**	Strongly disagree	3	18
	Disagree	3	18
	Neutral	4	24
	Agree	0	0
	Strongly agree	4	24
	Non-applicable	3	18
**Thanks to the information on the website, I think I have the knowledge and skills to support my child to solve possible (future) sexual problems.**	Strongly disagree	2	12
	Disagree	3	18
	Neutral	4	24
	Agree	3	18
	Strongly agree	3	18
	Non-applicable	2	12
**Thanks to information on the website, I feel able to support my child to improve their sexual health.**	Strongly disagree	1	6
	Disagree	4	24
	Neutral	5	29
	Agree	2	12
	Strongly agree	3	18
	Non-applicable	2	12
**Thanks to information on the website, I feel able to help my child solve (future) sexual problems.**	Strongly disagree	2	12
	Disagree	1	6
	Neutral	5	29
	Agree	5	29
	Strongly agree	2	12
	Non-applicable	2	12
PART C		***n***	**%**
**Is the information on the website presented clearly and comprehensibly?**	Yes	8	47
	Reasonably	6	35
	No	2	12
	I don't know	1	6
**Is the information on the website accurate?**	Yes	9	53
	Reasonably	2	12
	No	0	0
	I don't know	6	35
**Is the information on the website complete?**	Yes	1	6
	Reasonably	7	41
	No	4	24
	I don't know	5	29
**Are you aware of any additional resources that may be included on the website?**	Yes	2	12
	No	15	88
**Is the information on the website easy to find?**	Yes	10	59
	Reasonably	5	29
	No	1	6
	I don't know	1	6

(*Continued*)

 © 2025. The Author(s).

(*Continued*)

Parents of patients with ARM/HD			
**Is it clear who the website is intended for?**	Yes	15	88
	Reasonably	1	6
	No	0	0
	I don't know	1	6
**Is the layout appealing?**	Yes	7	41
	Reasonably	7	41
	No	3	18
	I don't know	0	0
**Are the images informative?**	Yes	8	47
	Reasonably	5	29
	No	1	6
	I don't know	3	18
**Is the font size and style on the website attractive to you?**	Yes	13	76
	Reasonably	4	24
	No	0	0
	I don't know	0	0
**Does the website have a reliable/professional appearance?**	Yes	10	59
	Reasonably	5	29
	No	1	6
	I don't know	1	6
**Is the website intuitive and easy to navigate?**	Yes	9	53
	Reasonably	6	35
	No	1	6
	I don't know	1	6
**Is the website applicable for you? (See www.dictionary.com/browse/applicable)**	Yes	13	76
	Reasonably	3	18
	No	0	0
	I don't know	1	6
**Would you recommend the website to patients with an anorectal malformation or Hirschsprung disease?**	Yes	11	65
	Probably	4	24
	Maybe	2	12
	No	0	0
	I don't know	0	0
**Would you recommend the website to parents of children with an anorectal malformation or Hirschsprung disease?**	Yes	11	65
	Probably	5	29
	Maybe	1	6
	No	0	0
	I don't know	0	0
**Would you recommend the website to healthcare providers?**	Yes	10	59
	Probably	2	12
	Maybe	3	18
	No	0	0
	I don't know	2	12

 © 2025. The Author(s).

(*Continued*)

Parents of patients with ARM/HD			
**Would you recommend the website to someone else?**	Yes	6	35
	Probably	2	12
	Maybe	7	41
	No	1	6
	I don't know	1	6
**Do you think anything needs to be improved on the website?**	Yes	8	47
	No	2	12
	I don't know	7	41
**Do you think that it would be helpful to have the website translated into your native language?**	Yes	16	94
	No	0	0
	I don't know	1	6
**Are there any cultural considerations that should be taken into account for the website?**	Yes	3	18
	No	1	6
	I don't know	13	76

[a]Percentages are rounded to the nearest whole number. As a result, they may not add up to 100%.

 © 2025. The Author(s).
